# Putting on the brakes: Bacterial impediment of wound healing

**DOI:** 10.1038/srep14003

**Published:** 2015-09-14

**Authors:** Kimberly M. Brothers, Nicholas A. Stella, Kristin M. Hunt, Eric G. Romanowski, Xinyu Liu, Jes K. Klarlund, Robert M. Q. Shanks

**Affiliations:** 1 The Charles T. Campbell Ophthalmic Microbiology Laboratory, UPMC Eye Center, Ophthalmology and Visual Sciences Research Center, Department of Ophthalmology (OVSRC), University of Pittsburgh School of Medicine, Pittsburgh, PA, USA; 2 Department of Chemistry, University of Pittsburgh, Pittsburgh, PA, USA

## Abstract

The epithelium provides a crucial barrier to infection, and its integrity requires efficient wound healing. Bacterial cells and secretomes from a subset of tested species of bacteria inhibited human and porcine corneal epithelial cell migration *in vitro* and *ex vivo*. Secretomes from 95% of *Serratia marcescens*, 71% of *Pseudomonas aeruginosa*, 29% of *Staphylococcus aureus* strains, and other bacterial species inhibited epithelial cell migration. Migration of human foreskin fibroblasts was also inhibited by *S. marcescens* secretomes indicating that the effect is not cornea specific. Transposon mutagenesis implicated lipopolysaccharide (LPS) core biosynthetic genes as being required to inhibit corneal epithelial cell migration. LPS depletion of *S. marcescens* secretomes with polymyxin B agarose rendered secretomes unable to inhibit epithelial cell migration. Purified LPS from *S. marcescens*, but not from *Escherichia coli* or *S. marcescens* strains with mutations in the *waaG* and *waaC* genes, inhibited epithelial cell migration *in vitro* and wound healing *ex vivo*. Together these data suggest that *S. marcescens* LPS is sufficient for inhibition of epithelial wound healing. This study presents a novel host-pathogen interaction with implications for infections where bacteria impact wound healing and provides evidence that secreted LPS is a key factor in the inhibitory mechanism.

The cornea, a transparent tissue at the front of the eye, is a useful model for studying the general processes of wound healing due to its transparency and has similar healing characteristics to other tissues^1^. Corneal wound healing problems are closely related to the inability to reform a complete and well-attached epithelium which leaves the deeper cell layers of the cornea vulnerable to bacterial infection^2^. For example, *Pseudomonas aeruginosa*, an important ocular pathogen, has increased adherence to wounded compared to intact corneal tissue when assessed *ex vivo*^3^ and *in vivo*^4^. Conversely, the loss of corneal epithelium is associated with bacterial keratitis suggesting that bacteria induce erosion of the corneal epithelium and prevent it from healing^5^. Pathogenic bacteria can invade the corneal stroma, release destructive enzymes that damage the stroma, and induce ulceration^6^. In most corneal wound studies, bacteria are associated with wound healing complications^7^. Beyond the cornea, the impact of bacteria on chronic wounds is poorly understood. Despite this, few studies have explored the impact of bacteria on wound healing and the mechanisms by which they can manipulate the healing process.

In the present study, we sought to evaluate whether bacteria that commonly infect ocular tissues or cause nosocomial infections are able to alter corneal wound healing and to determine the bacterial mechanism by which they inhibit corneal epithelial wound healing. Results from these studies demonstrate secretomes from different bacterial genera inhibit corneal epithelial wound healing. In the case of *S. marcescens*, evidence suggests that LPS is necessary and sufficient for healing inhibition.

## Results

### Inhibition of epithelial cell migration *in vitro* by bacterial secretomes

**Figure 1:**
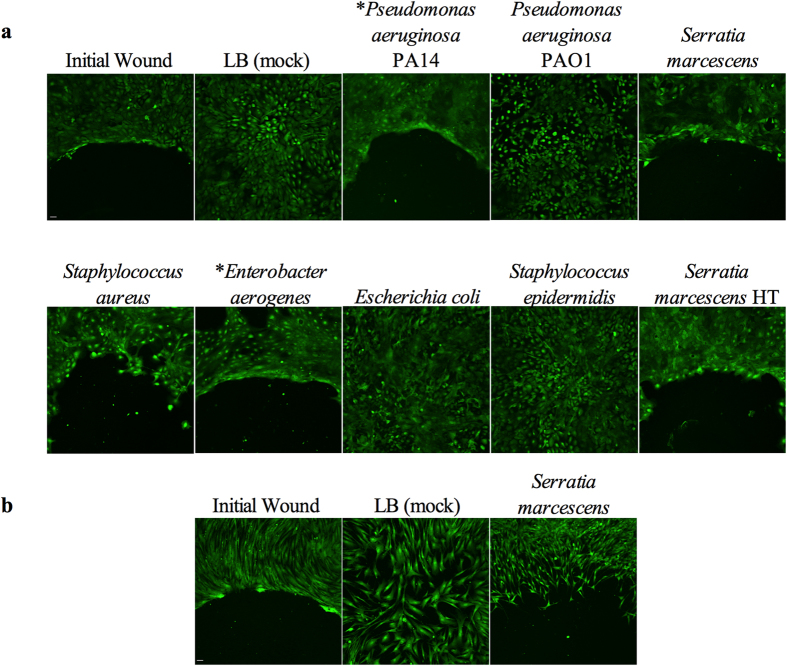
Inhibition of cell migration *in vitro* by some bacterial secretomes. (**a**) Images of Calcein AM stained HCLE cells treated with secretomes. *OD_600_ = 1.0 secretomes used in the shown experiment. HT = secretome incubated at 95 °C for 10 minutes. (**b**) Images of Calcein AM stained human foreskin fibroblast cells treated with LB (mock) and *Serratia marcescens* PIC3611 secretomes. Initial wound = cells incubated in the presence of a silicone stopper to determine size of original wound. Image taken is half of wound area. Scale bar = 50 μm.

*In vitro* cell migration assays with stratified layers of human corneal limbal epithelial (HCLE) cells were used to test whether secretomes, secreted and shed molecules, inhibited corneal epithelial cell migration. Since *P. aeruginosa* and *S. marcescens* are the most common causes of contact-lens associated keratitis and are commonly isolated from chronic wounds^8^, we tested a panel of *P. aeruginosa* and *S. marcescens* strains used in laboratory research and derived from clinical keratitis for the capacity to prevent corneal epithelial cell migration. For each tested strain, the cell layer either completely filled in the gap to an extent similar to the LB-challenged negative control (no inhibition) or exhibited virtually no movement over the 24 h course of the experiment (inhibited corneal epithelial cell migration) (Fig. 1 and Supplementary Fig. S1).

Two commonly used *P. aeruginosa* strains yielded surprisingly different outcomes. Strain PA14^9^, but not PAO1^10^ inhibited corneal epithelial cell migration (Fig. 1a). Commonly used research strains of *S. marcescens* PIC3611, Db11^11^, NIMA^12^, and environmental isolate CHASM^13^, all inhibited corneal epithelial wound healing (Fig. 1a and Supplementary Fig. S1). Interestingly, secretomes from neonatal intestinal isolate UC1SER^14^ killed HCLE cells at the full dose, but failed to inhibit cell migration at the half dose (Supplementary Fig. S1).

Secretomes from 15 out of 16 (94%) of the tested keratitis strains of *S. marcescens* inhibited HCLE cell migration (Supplementary Fig. S1). Four out of five (80%) of *P. aeruginosa* keratitis strains inhibited HCLE cell migration and 2 out of 7 (29%) *S. aureus* strains inhibited HCLE cell migration (Fig. 1a and Supplementary Fig. S1). Based on Calcein AM staining several of the keratitis strains were cytotoxic when 500 μl of normalized secretome was added to the wells, but inhibited migration without killing the HCLE cells when used at 25 μl per well (Supplementary Fig. S1).

A number of bacterial genera associated with contact lens case contamination, ocular infection and other human disease were also tested. Secretomes from one strain of *Citrobacter freundii* and one of four clinical isolates of *Enterobacter aerogenes* inhibited HCLE cell migration (Fig. 1a). *Acinetobacter baumanii* (n = 5 tested strains)*, Achromobacter xylosoxidans* (n = 1)*, Escherichia coli* K746 and MC4100 (n = 2), *Klebsiella pneumoniae* (n = 1), and *Stenotrophomonas maltophilia* (n = 1) did not inhibit HCLE migration. A single strain of *Enterococcus faecalis* and *Staphylococcus epidermidis* failed to inhibit wound healing. *S. marcescens* PIC3611 was used as a model organism to study bacterial influence on corneal epithelial cell migration for the remainder of this study.

Physical wounding of stratified HCLE cells creates a different kind of wound than gaps made with a silicone stopper, as the cellular contents of the damaged HCLE cells are released into the medium which can activate additional signalling pathways^15^. Treatment of physical wounds with *S. marcescens* secretomes resulted in inhibited corneal cell migration (Supplementary Fig. S2). Secretomes from *S. marcescens* also effectively inhibited migration of human fore skin fibroblast cells (Fig. 1b).

### Inviable and viable *S. marcescens* inhibit corneal epithelial cell migration *in vitro*.

Live and dead bacteria were tested for their ability to inhibit corneal cell migration since it is expected that contact lenses can bring these into contact with the corneal epithelium. *S. marcescens* ~2 × 10^5^ CFU, both live or killed using antibiotic and heat treatment, were capable of inhibiting corneal cell migration in a manner similar to the secretomes (Supplementary Table S2).

### *S. marcescens* inhibits corneal epithelial cell migration *ex vivo*

**Figure 2:**
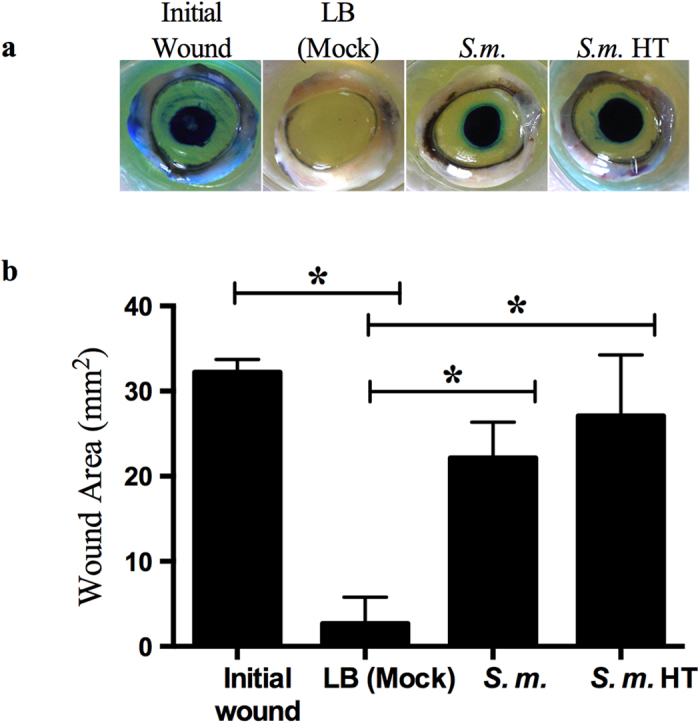
*S. marcescens* (*S.m.*) secretomes inhibit corneal wound healing *ex vivo.* LB (mock) (n = 6) and secretomes (n = 6) were added onto wounded corneas and incubated for 48 hours. To observe epithelial defects the corneal tissue was stained with Richardson solution (blue stain). Initial wounds (n = 3) are corneas wounded and stained at end of experiment to determine original wound size. HT = secretome incubated at 95 °C for 10 minutes (n = 6). (**a**) Representative images of porcine corneas treated with secretomes. (**b**) Measurements of corneal wounds from *ex vivo* corneal organ culture. Error bars represent one standard deviation. *p < 0.05 by Tukey’s post hoc analysis.

To determine if the wound inhibitory migration phenotype occurs with intact mammalian tissue, we used a porcine corneal organ culture epithelial wound healing model^16^,^17^. Mechanically wounded corneas did not heal after challenge with *S. marcescens* secretomes, whereas control LB (mock) treatments healed (Fig. 2) recapitulating results from the *in vitro* experiments. Thus, bacterial inhibition of wound healing also occurs *ex vivo* with a complex multicellular tissue.

### *S. marcescens* secretomes do not kill HCLEs or inhibit HCLE cell attachment to plastic

**Figure 3:**
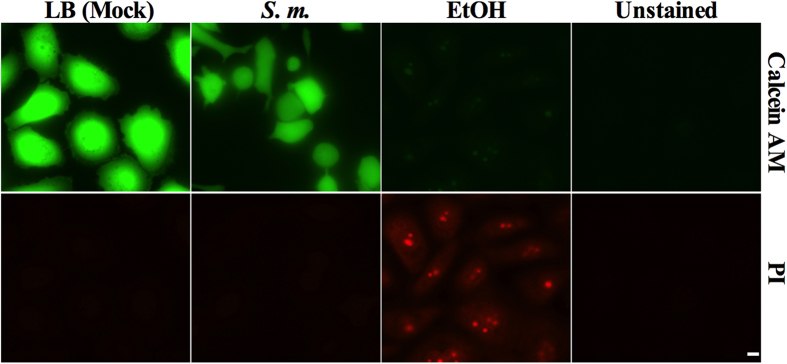
*S. marcescens* secretomes are not cytotoxic to corneal epithelial cells. LB (mock) and secretomes were added to HCLEs and incubated overnight. Ethanol treatment was used as a positive control for inviable cell staining. Cells were stained with 0.5 μM Calcein AM and 1 μM propidium iodide (PI). Unstained corneal epithelial cells were imaged to verify there was no background fluorescence. Scale bar = 10 μm.

To test whether inhibition of epithelial migration was due to cell death, we stained bacterially challenged and control HCLE cell layers with fluorescent stains that differentiate living (Calcein AM) and dead (propidium iodide, PI) cells (Fig. 3). HCLE cells treated with LB medium or bacterial secretomes did not reveal any changes in cell viability, whereas ethanol-treated HCLEs showed robust PI staining and loss of Calcein AM fluorescence indicating cell death (Fig. 3). Similar results were observed when cytotoxicity was determined by Alamar Blue (Supplementary Fig. S3a).

To test whether decreased migration was due to inability of HCLE cells to attach to surfaces, we quantified HCLE attachment to tissue culture treated plastic under two conditions: 1) wells were coated with secretomes by desiccation and 2) secretomes were added directly to the KSFM growth medium. No differences in cell attachment to plates was observed (Supplementary Fig. S3b p = 0.92, and S3d p = 0.18) indicating the wound inhibitory factor (WIF) does not influence cell migration by interfering with cell attachment. Together these data suggest that *S. marcescens* WIF does not prevent HCLE migration by killing corneal cells or by preventing their attachment to plastic.

### *S. marcescens*-treatment of HCLEs alter the actin cytoskeleton

**Figure 4:**
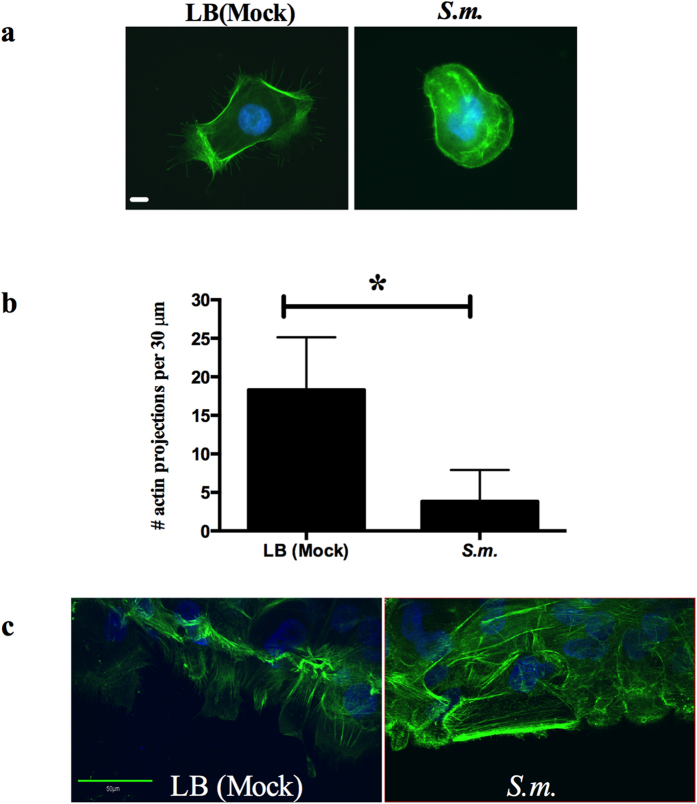
*S. marcescens* secretomes alter HCLE actin cytoskeleton. (**a**) LB (mock) and secretomes were added to HCLEs and incubated for 4 hours. HCLEs were stained with Alexa-488 phalloidin for actin (green) and Hoechst 33342 for DNA (blue) and imaged. Scale bar = 10 μm. (**b**) Actin projections per 30 μm cell area were quantified (LB n = 36, *S.m.* n = 40). Error bars represent standard deviation. *p < 0.05 by Student’s T test. (**c**) Stratified HCLEs were treated with LB (mock) and secretomes for 3 hours. Cells were fixed and stained as described above. The center of the “wound” was imaged by confocal microscopy. Scale bar = 50 μm.

Several species of bacteria have demonstrated the ability to alter the mammalian actin, cytoskeletal components that are necessary for cell migration^18^. We examined whether the actin cytoskeleton was changed in response to *S. marcescens* secretomes. Fewer actin projections were counted on isolated HCLE cells that had been treated with *S. marcescens* secretomes (Fig. 4a,b) compared to HCLE cells treated with LB (mock). In epithelial cell migration experiments, LB (mock) treated HCLEs started to reorient themselves into the area formerly occupied by the agarose stopper, whereas HCLEs treated with *S. marcescens* secretomes showed no reorientation into the area, and had no projections extending from the leading edge (Fig. 4c). Thus, treatment with *S. marcescens* secretomes is associated with pronounced alterations of the actin cytoskeleton.

### Biochemical analysis of WIF

To characterize WIF, we conducted a battery of biochemical analyses. The pH of *S. marcescens* secretomes (7.1 +/− 0.29) did not differ substantially from LB (7.4 +/− 0.59) ruling out pH differences of the secretomes as the cause of the wound inhibitory phenotype.

To determine if WIF was heat labile, secretomes were heat-treated under two different temperature conditions, 65 °C for 1 hour, or 95 °C for 10 minutes, cooled on ice, and tested for inhibition of HCLE cell migration and wound healing *ex vivo* (Table S2 and Figs 1A and 2). Heat treatment had no effect on the wound inhibitory phenotype suggesting that WIF is not a protein.

To determine the effect of freezing on inhibition of corneal cell migration, *S. marcescens* secretomes were frozen at −20 °C and −80 °C, thawed, and tested. Samples thawed only once were found to effectively inhibit corneal epithelial cell migration, whereas samples subjected to multiple freeze-thaws lost their ability to inhibit corneal epithelial cell migration (Table S2) indicating WIF is freeze-thaw susceptible as has been shown for molecules such as lipolysaccharide (LPS)^19^.

Chloroform extraction of *S. marcescens* secretomes was performed to determine WIF’s relative polarity. The aqueous fraction was effective at inhibiting corneal cell migration, whereas the chloroform fraction was not (Supplementary Table S2). WIF is a polar molecule as it was soluble in the polar aqueous phase rather than the non-polar solvent phase.

Ion exchange chromatography of secretomes was performed with 1) hydroxylapatite (HA), a form of calcium phosphate that can be used as a chromatography matrix^20^, and 2) with HP-20 diaion, a polyaromatic resin of hydrophobic compounds that binds lipopolysaccharide^21^, antibiotics, and other biomolecules. Flow-through fractions of HP-20 columns did not inhibit cell migration (Supplementary Fig S2 and Table S2), and methanol extraction of the secretome-incubated HP-20 resin released the inhibitory factor indicating that it binds to HP-20 (Table S2 and Fig. S2). Hydroxylapatite did not bind to the inhibitory factor sufficiently to prevent the flow-through fraction from inhibiting HCLE cell migration (Table S2).

*S. marcescens* secretomes were subjected to chemical and enzymatic analysis according to Karwacki *et al.*^22^. Samples were treated with DNase, RNase, lipase from porcine pancreas, hyaluronidase^23^, and serralysin-family metalloprotease protease inhibitor AprI^24^, along with controls to verify enzyme activity. None of these treatment conditions had an effect on the ability of secretomes to inhibit epithelial cell migration (Supplementary Table S2). Additionally, the *S. marcescens* PIC3611 secretome contains metalloprotease, lipase/esterase, and nuclease activities^25^ indicating that WIF is not inactivated by native secretome enzymes.

To estimate the molecular weight of *S. marcescens* WIF, secretomes were centrifuged in different molecular weight cutoff (MWCO) spin columns. The column retentate from 3000, 10,000, and 20,000 MWCO columns was able to inhibit HCLE cell migration, whereas only the flow through of the 30,000 MWCO column inhibited corneal epithelial cell migration (Supplementary Table S2). The molecular weight of WIF is therefore estimated to be in the range of 10–30 kDa.

### Genetic analysis implicates the LPS biosynthetic locus in inhibition of HCLE cell migration

**Figure 5:**
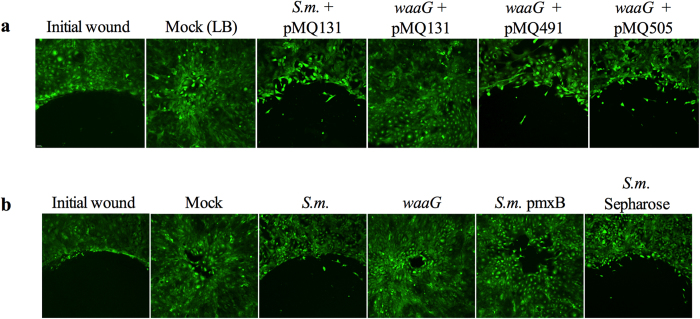
*S. marcescens* secretomes from a mutant in the LPS biosynthetic locus transposon mutant (*waaG*) and LPS depleted secretomes do not inhibit cornea cell migration. LB (mock) and secretomes were added to HCLEs and incubated for 18–24 hours. (**a**) HCLE cell migration assays treated with secretomes from *S.m.* (pMQ131 vector control), LPS transposon mutant (*waaG* pMQ131 vector control), pMQ491 (*waaG* alone), and pMQ505 (*waaG* and *orf*10). (**b**) HCLEs treated with mock and secretomes from *waaG* transposon mutant (*waaG*), LPS depleted (pmxB), and agarose bead control treated secretomes (*S.m.* sepharose). Scale bar = 50 μm.

To identify bacterial genes involved in making or regulating WIF we generated and screened a transposon mutant library and a selection of previously defined mutants from our collection of mutant strains for a loss of WIF. Of 1134 tested transposon mutants, several were isolated with a failure to inhibit corneal epithelial cell migration. These included two genes that had a moderate effect on WIF activity, a *S. marcescens degS* homolog, predicted to code for a periplasmic protease and outer membrane envelope stress regulator, and *gidA*, a glucose inhibited division protein A gene. Mutations in four different genes conferred an almost complete loss of WIF activity. These mutations mapped to a predicted two-component system histidine-kinase *eepS*^26^, *hfq*, that codes for RNA-stability regulator, and mutations in two genes in the LPS biosynthetic locus *waaC* and *waaG* (Fig. 5). LPS genes one in *waaC* and two in *waaG* designated as ORF 3 and ORF 9 according to Coderch *et al.*, have been predicted to influence the core structure of LPS^27^,^28^.

### Complementation of the *S. marcescens* *waaG* mutant restores WIF phenotype

**Figure 6:**
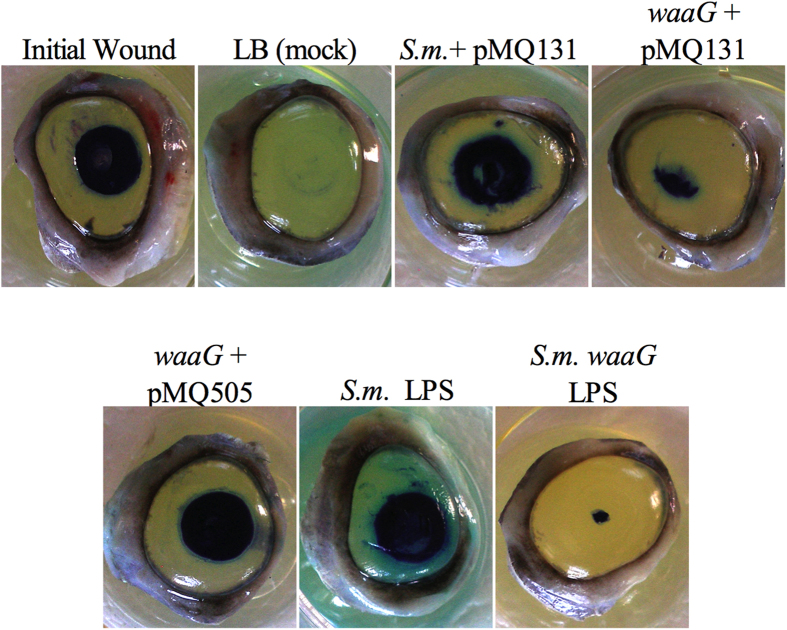
Purified LPS from *S. marcescens* (*S.m.*) PIC3611 inhibits corneal wound healing *ex vivo*. *waaG* secretomes and 10000 ng/ml *waaG* purified LPS (n = 3) do not inhibit corneal wound healing and complementation with pMQ505 (*waaG* and *orf*10) restores wound healing inhibition. LB (mock) (n = 4), secretomes (n = 6), and purified LPS were added dropwise onto wounded corneas and incubated for 48 hours. Initial wounds (n = 2) are corneas wounded and stained at end of experiment to determine original wound size. To determine remaining wound size corneas were stained with Richardson solution (blue stain).

To genetically validate that the *waaG* LPS biosynthesis gene is necessary for WIF, complementation analysis was performed. The *waaG* gene is the second gene in a three-gene operon consisting of *waaQ*, *waaG*, and *orf10*^27^. Therefore, a transposon mutation in *waaG* would likely have a polar effect on *orf10* expression. The *waaG* gene alone, as well as *waaG* with *orf10* were cloned into a multicopy plasmid under transcriptional control of the *P*_*lac*_ promoter generating pMQ491 and pMQ505 respectively. Both plasmids restored inhibition of corneal epithelial cell migration to the *waaG* mutant strain, whereas the negative control vector (pMQ131) did not (Fig. 5a). These results were replicated using *ex vivo* corneal organ culture (Fig. 6) indicating the mutation in LPS biosynthetic gene *waaG* alone was responsible for the loss of WIF, strongly implicating LPS as a candidate molecule for WIF.

### *S. marcescens* secretomes treated with Polymyxin B agarose are unable to inhibit corneal cell migration *in vitro*

In order to test the prediction that *S. marcescens* LPS is WIF (or requires LPS), secretomes were incubated in the presence of polymyxin B agarose. Polymyxin B binds to LPS and is often used to remove LPS from liquids, fluids, and protein preparations^29^. *S. marcescens* secretomes treated with polymyxin B agarose had significantly reduced concentrations of LPS (Supplementary Fig. S4) and, crucially, failed to inhibit HCLE cell migration, providing additional evidence that LPS is necessary or required for wound inhibition (Fig. 5b).

### Purified *S. marcescens* LPS, but not *E. coli* LPS inhibits corneal epithelial cell migration *in vitro*

As noted above, *E. coli* secretomes failed to inhibit epithelial cell migration even though *E. coli* has LPS. To test whether *E. coli* releases lower levels of LPS, supernatants from cultures of *E. coli* and *S. marcescens* were analyzed for LPS and contained 25,140 ng/ml and 18,814 ng/ml, respectively (Supplementary Fig. S5), indicating that reduced LPS shedding was not responsible for the difference between species.

To test whether LPS molecules are sufficient for corneal epithelial wound inhibition rather than due to other secreted factors, we isolated and purified LPS from *S. marcescens* wild-type, *waaC* and *waaG* mutant cultures, and *E. coli*^30^,^31^. Equal concentrations of *S. marcescens* and *E. coli* LPS were tested for HCLE cell migration inhibition.

**Figure 7:**
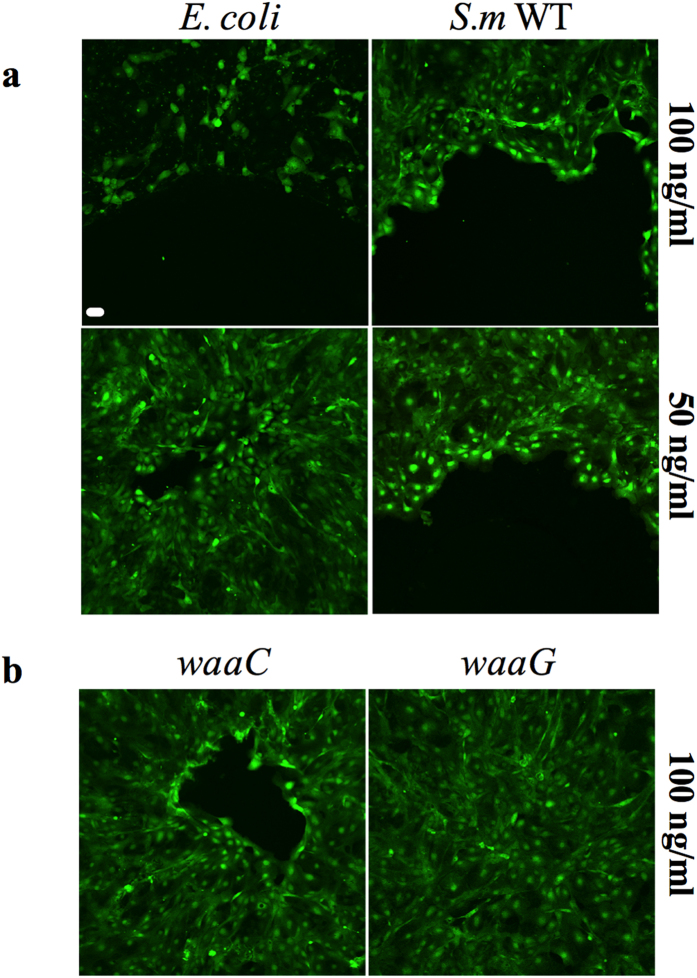
*S. marcescens* (*S.m.*) PIC3611 LPS, but not LPS derived from *E. coli* or *S.m. waaC* and *waaG* mutants, inhibits corneal cell migration *in vitro*. LPS was purified from *E. coli* K746*, S. m.* WT, *waaC* and *waaG* LPS mutants. Scale bar = 50 μm. (**a**) *E. coli* K746 and *S. m.* LPS cell migration experiments. Loss of staining indicates cell death or removal of corneal cells from surface. **(b**) *waaC* and *waaG* LPS cell migration experiments.

Unlike *S. marcescens* LPS, higher concentrations (100 ng/ml) of *E. coli* LPS were cytotoxic to HCLEs as observed by a loss of Calcein AM viability staining (Fig. 7a). Wild-type *S. marcescens* PIC3611 LPS inhibited HCLE cell migration at concentrations of 50 ng/ml to 55868 ng/ml without being cytotoxic, whereas *waaC* and *waaG* mutant derived LPS at 100 ng/ml), and *E. coli* LPS at 50 ng/ml did not (Fig. 7) providing evidence that *S. marcescens* LPS is sufficient for wound inhibition. This result also suggests a difference in the LPS structure rather than the amount shed by the two different organisms.

## Discussion

The cornea is a relevant and useful model to study the impact of bacteria on wound healing. Integrity of the cornea is critical for vision as well as proper clearance of bacteria. The use of contact lenses promotes exposure of the ocular surface to bacteria^32^,^33^, and due to the large number of contact lens wearers worldwide (~60 million), corneal infection is an increasing problem^32^,^34^. Also, corneal trauma can initiate sight-threatening invasive bacterial infections^35^,^36^.

We hypothesized that bacterially derived factors modulate epithelial cell behavior, specifically corneal epithelial wound healing. The impact of bacterial secreted factors on corneal epithelial wound healing was tested using *in vitro* and *ex vivo* models. The data presented here demonstrate that several opportunistic pathogens including major ocular pathogens *P. aeruginosa*, *S. marcescens*, and *S. aureus* produce wound inhibitory factors. *S. marcescens* inhibition of epithelial cell migration was also observed using human foreskin fibroblasts indicating that this phenotype is not cornea or epithelial cell specific. WIF activity was most commonly observed in *S. marcescens* and *P. aeruginosa* isolates highlighting the clinical importance of studying these ocular pathogens. Other bacteria with WIF include the important ocular and nosocomial pathogens *C. freundii* and *E. aerogenes*. In contrast, several other tested Gram-negative bacteria did not inhibit wound healing. To our knowledge, this is the first study to characterize bacterial inhibiton of corneal epithelial cell migration and wound healing.

While undiluted supernatants from some clinical isolates were toxic, *S. marcescens* did not cause cytotoxicity to HCLE cells and had no effect on attachment of HCLE cells to a plastic surface, suggesting that toxicity or effects on adhesion were not responsible for migration inhibition. TUNEL staining suggested that apoptosis or other forms of cell death^37^ was not responsible for inhibition of wound healing in porcine corneas *ex vivo*.

Our studies of *S. marcescens* secretome-treated HCLEs revealed a reduced number of actin projections compared to the LB (mock) control, as well as a failure of stratified HCLEs to orient into the “wound” area 3 hours after removal of the agarose barrier, although actin stress fibers were clearly visible. These results indicate that *S. marcescens* secretomes profoundly affect the actin cytoskeleton. These results differ from other studies in human corneal cells treated with *P. aeruginosa* that resulted in a dramatic loss of the actin stress fibers^38^.

LPS is a biologically active glycosylated phospholipid present on the outer leaflet of Gram-negative bacterial cell membranes. LPS from *Salmonella typhi* and *Esherichia coli* have the ability to change cell morphology and actin organization as well as promoting migration in monocytes^39^. Studies by Chakravortty *et al.* show bovine aortic endothelial cell rounding, cytoskeletal disorganization and alteration in the actin cytoskeleton when treated with *E. coli* LPS^40^ consistent with our observations of alterations in the HCLE actin cytoskeleton.

The structure of LPS is variable among bacterial species and even between strains of the same species^41^,^42^,^43^. The structure of LPS can impact host-pathogen interactions. In fact, some bacteria evade the immune system by modifying their LPS, thereby altering susceptibility to antimicrobial peptides and interactions with TLR4/MD2^44^,^45^. Since many of the tested organisms that do not inhibit wound healing have LPS, it appears that that only specific LPS structures are capable of inhibiting cell migration.

Transposon mutagenesis of the *S. marcescens* genome implicated the lipolysaccharide (LPS) biosynthetic locus as having a role in the inhibitory phenotype. We restored inhibition of HCLE migration by complementation of the *waaG* mutant, indicating that the *waaG* mutation rather than an unknown mutation or a polar effect was responsible for the loss of WIF. Together these results provide genetic evidence for LPS in inhibition of corneal cell migration and suggest that the LPS core or O-antigen is necessary for migration inhibition.

Biochemical data also supported a role for LPS as WIF. When wild-type *S. marcescens* secretomes were depleted for LPS using polymyxin B agarose, a loss of inhibition of HCLE cell migration and a reduction in LPS concentration was observed. HP-20 resin, which binds LPS^21^, bound WIF, whereas hydroxylapatite, which does not efficiently bind LPS^21^, did not remove WIF from secretomes (Supplementary Table S2). Size fractionation, freeze-thaw sensitivity, polymyxin B agarose depletion, and other biochemical analysis were consistent with the identification of WIF as LPS^19^,^46^. Only LPS purified from wild-type *S. marcescens* but not from LPS mutants or *E. coli*, was determined to be sufficient for inhibition of HCLE cell migration and corneal wound healing.

Treatment of cells with LPS *in vitro* and *in vivo* has been shown to have a variety of effects on corneal ulcers, cell migration, and wound healing. Topical application of 10,000 ng *S. marcescens* LPS after ocular abrasion resulted in more severe corneal ulcers compared to treatment with same dose of *P. aeruginosa* LPS^47^. Studies by Kostarnoy *et al.* showed that topical application of *Salmonella typhi* LPS (~5 EU/mg) promoted wound healing in mice^48^. When rats with gastric ulcers were administered different doses of *E. coli* LPS by parenteral injection, a dose dependent inhibition of healing with maximal inhibition at 5 × 10^3^ EU/kg LPS was observed^49^.

Strikingly different from what was reported in this study with *S. marcescens* LPS, Eslani *et al.* demonstrated *E. coli* LPS (100 ng/ml) accelerated HCLE cell migration *in vitro*^50^. A similar enhanced migration phenotype was observed with human pulmonary mucoepidermoid carcinoma cell lines treated with 10,000 ng/ml LPS from *P. aeruginosa* serotype 10 ATCC 27316 LPS, whereas treatment with a higher dose (500,000 ng/ml) resulted cell migration inhibition^51^. However, studies by Loryman and colleagues showed opposite effects with a lower dose (1000 ng/ml) *P. aeruginosa* serotype 10 LPS inhibiting human epidermal keratinocyte migration *in vitro*^52^ indicating LPS inhibition of cell migration may be different depending on the cell line. LPS of an unspecified species at 100 and 50,000 ng/ml inhibited intestinal enterocyte migration *in vitro*^53^. Our present study demonstrates the ability of *S. marcescens* LPS to fully inhibit corneal cell migration at doses of 100 ng/ml complementing studies by Cetin *et al.*, whereas *E. coli* LPS at the same concentration was cytotoxic to HCLE cells and importantly ineffective at inhibition at lower doses. Our studies implicate *S. marcescens* LPS in inhibition of corneal epithelial wound healing with the ability to inhibit wound healing at doses as low as 50 ng/ml.

Currently it is not clear whether *P. aeruginosa* and other tested organisms inhibit cell migration via LPS. Clearly, LPS is not the only way bacteria can inhibit corneal cell migration, as *S. aureus* does not have LPS and was able to inhibit cell migration. This suggests that bacteria have evolved a number of mechanisms that impact cell migration behavior.

In summary, we have found that several relevant opportunistic pathogens can inhibit cell migration and ocular wound healing, and our evidence supports that *S. marcescens* LPS is an inhibitory factor of corneal wound healing. These studies indicate a role for bacterial secreted factors in wound healing inhibition and suggest the potential use of LPS depletion as a therapeutic strategy to prevent infection and enhance wound healing.

## Methods

### Bacterial growth and media

Bacteria (Table S1) stored at −80 °C in glycerol frozen stocks were streaked to single colonies on LB agar (0.5% yeast extract, 1% tryptone, 0.5% NaCl, 1% agar) and grown at 30 °C. Overnight bacterial cultures were prepared by inoculating a single colony of bacteria into 5 ml LB broth (0.5% yeast extract, 1% tryptone, 0.5% NaCl) and incubating overnight (18–20 h) at 30 °C with shaking. Keratitis and endophthalmitis strains were isolated at the Charles T. Campbell Laboratory of Ophthalmic Microbiology at the University of Pittsburgh Eye Center and kindly provided by Regis P. Kowalski. *Enterobacter aerogenes* and *Klebsiella pneumoniae* clinical isolates were kindly provided by Cornelius Clancy, and Minh-Hong Nguyen from the Division of Infectious Diseases at the University of Pittsburgh.

### Preparation of bacteria-free conditioned media

Secretomes (stationary phase culture supernatants with bacteria removed) were prepared by growing cultures of bacteria (Supplementary Table S1) as noted above. Cultures were normalized by dilution to OD_600_ = 2.0 with LB broth and bacteria were removed by centrifugation at 16,000 × g and the supernatant filtered through a 0.22 μm PVDF (Millex #SLGV033RS) filter.

### *In vitro* cell migration experiments

*96 well plate assays:* Human corneal limbal epithelial (HCLE) cells^54^ were cultured in keratinocyte serum-free medium (KSFM) (Gibco Catalog number 10724–011) containing 100 μg/ml penicillin, 100 μg/ml streptomycin (Corning #30-002-CL), 0.2 ng/ml embryonic growth factor (EGF) (Gibco #10450-013), and 25 μg/ml bovine pituitary extract (Gibco #13028-014). HCLEs were seeded into 96 well plates containing a silicone “stopper” (ORIS™ Platypus Technologies, LLC #CMAU101) at a density of 10^4^ cells per well. HCLE monolayers were rinsed with phosphate buffered saline (PBS), pH 7.4, and supplemented with “stratification medium” consisting of Dulbecco’s modified eagle’s medium (Cellgro #10-017-CV), and F-12 medium (Bio-Wh. 12-615F) at a 1:1 ratio, supplemented with 10% newborn calf serum (Atlanta Biologicals #S11995), 10 ng/ml EGF, 100 μg/ml penicillin, and 100 μg/ml streptomycin and grown for 3 days. The cell layers were washed 3 times with PBS and incubated with 100 μl stratification medium. LB (mock) and 50 μl secretomes were added to stratification medium. HCLE cells were incubated at 37 °C + 5% CO_2_ for 18–24 hours. After incubation with secretomes or controls, HCLE cells were rinsed with PBS and stained with 0.5% crystal violet and formaldehyde (0.02%) or with 0.5 μM Calcein AM (Invitrogen catalog number C3099) for 15 minutes.

*12 well plate assays:* HCLE cells were seeded in KSFM medium in 12 well plates (Costar #3513) with no stopper at a density of 1.2 × 10^4^ cells per well and stratified as described above. Each well was “wounded” with an Amoils epithelial scrubber (Innovative Excimer solutions) using a sterile 6.5 mm diameter brush head and treated with secretomes (500 μl into 1 ml stratification medium).

*HFF cell migration assays:* Human foreskin fibroblasts (HFF) were grown in DMEM containing 10% fetal bovine serum (Atlanta Biologicals #S11150), 100 μg/ml penicillin, and 100 μg/ml streptomycin in 96 well plates containing silicone stoppers as described above. Secretomes were added to HFF cells at the same dose as described above and allowed to migrate for 24–48 hours then stained with 0.5 μM Calcein AM for 15 minutes.

*Imaging of cell migration assays:* Cell layers were imaged on an Olympus Fluoview FV-1000 laser scanner confocal microscope with a 10x (0.3 NA) objective and analyzed with Fluoview image viewing software version 3.1.

### Ex vivo wound healing model using porcine corneas

A corneal organ culture wound healing model was used based on those of Foreman *et al.*^16^ and Xu *et al.*^17^. Porcine eyes were obtained from Sierra Medical (Whittier, CA) within 24 hours of harvesting. Eyes were placed in sterile phosphate buffered saline containing 100 μg/ml penicillin, 100 μg/ml streptomycin, 100 μg/ml gentamicin, and 2.5 μg/ml amphotericin B. Epithelial defects were introduced into porcine corneas with an Amoil’s epithelial scrubber using a sterile 6.5 mm diameter brush. Wounded corneas and approximately 4 mm of surrounding sclera were excised with Vannas scissors and placed epithelium side up onto molds of minimal essential medium (MEM) (Gibco #41500-018), 1000 μg/ml collagen (rat tail, Sigma #C3867), and 1% agarose. MEM was placed in each well containing corneas until it covered up to the limbus leaving the cornea exposed to air. Mock (LB) and secretomes (1.5 ml) were added to 3 ml MEM, mixed and added drop wise on to corneas. Corneas were incubated at 37 °C + 5% CO_2_ for 48 hours and stained with Richardson solution (1% Azure II, 1% methylene blue, 1% borax). Corneas were digitally photographed from a fixed distance. Wound areas in mm^2^ were calculated in ImageJ (NIH) and graphed using GraphPad Prism version 6.0.

### Histology

Immediately at the endpoint of *ex vivo* experiments, porcine corneas were placed in 10% formalin for a minimum of 24 hours and sent to University of Pittsburgh Research Histology Services for Hematoxylin and Eosin (H&E) and TUNEL staining. TUNEL staining was conducted with a Millipore (#S7100) ApopTag peroxidase *in situ* apoptosis detection kit according to manufacturer’s protocols. As a positive control for cell death for TUNEL staining, corneas were exposed to UV light (120,000 microjoules) in a UV stratalinker (Stratagene model #2400). Images were captured with a 10x (0.3 NA) objective on an Olympus BX60 microscope with a SPOT camera model #2.3.1 and with SPOT software version 4.6.

### Cell viability assays

Cytotoxicity assays were performed using Alamar Blue viability reagent (Invitrogen #DAL1025) according to Wingard *et al.*^55^. Experiments were conducted in triplicate a minimum of three times. Calcein AM and propidium iodide viability analysis imaging were performed as the Alamar Blue Assay, but evaluated by microscopy. HCLE cells were grown to 40% confluence in KSFM media and cell layers were washed three times in PBS. A 30 minute exposure to 70% ethanol was used as a positive control for cell death. After incubation with secretomes or controls, HCLE layers were stained with 0.5 μM Calcein AM and 1 μM propidium iodide (Invitrogen #L7012) to detect cells with compromised membranes (dead) and incubated for 15 minutes at 37 °C + 5% CO_2_. After staining, HCLEs were washed three times in PBS and and supplied with KSFM media. Samples were imaged with a 40x (1.30 NA) objective on a Nikon TE2000-E microscope equipped with a Photometrics Cool Snap HQ camera. Images (n ≥ 10 fields per group) were captured using NIS-Elements 3.1 software Unstained wells of HCLEs (10 fields) were used as a control for background fluorescence. Experiments were performed on at least two different days in duplicate wells.

### HCLE attachment assays

To test whether secretomes inhibit HCLE cell attachment to plastic, wells were pretreated with secretomes followed by attachment, or co-incubated with secretomes during attachment. Five-hundred microliters of LB or secretomes were dried by desiccation in a laminar flow hood for 2 hours onto the bottom wells of a 12-well tissue culture plate. As a second assay, HCLEs were seeded into KSFM medium-containing secretomes at an “equivalent dose” used in migration assays (500 μl into 1 ml KFSM). HCLEs were seeded into control, LB (mock), and secretome treated wells at a density of 8.1 × 10^4^ cells per well. HCLEs were incubated at 37 °C + 5% CO_2_ for four hours, then imaged on a Nikon TE2000-E microscope with a 10X phase 0.30 NA objective as described above. Images were taken and attached cells were counted (n ≥ 18 cells per group) by three masked individuals and averaged. The experiment was repeated a minimum of two different times.

### Fluorescent actin staining

To observe individual cells, HCLEs were grown to ~30–50% confluence in KSFM media, treated with secretomes for 4 hours, and fixed in 4% paraformaldehyde for 10 minutes at room temperature. Fixed cells and cell layers were permeabilized and stained with Alexa Fluor 488 phalloidin (Molecular probes #A12379) according to manufacterer’s protocols. Cells were then incubated in 12.5 μM Hoechst 33342 (Invitrogen #62249) for 10 minutes to stain nuclei. Stained cells were mounted onto slides with ProLong Gold antifade reagent (Invitrogen #P36930). Samples were imaged with a 40 × 1.30 NA objective on a Nikon TE2000-E microscope as described above. Actin projections in individual cells were counted per a 30 μm segment of the cell edge for 36 cells per treatment group for LB and 40 cells per treatment group for WT and graphed using Graph Pad Prism 6.0. To observe actin in migrating cell layers, HCLEs were stratified in 12-well MatTek (#P12G-1.5–14-F) glass bottom dishes in the presence of an agarose strip barrier according to Block *et al.*^15^. The strip was removed and HCLEs were allowed to migrate for 3 hours into the cell-free zone left by the agarose strip then fixed in 4% paraformaldehyde and stained as described above and imaged on an Olympus Fluoview FV-1000 laser scanner confocal microscope with a 60X oil objective and analyzed with Fluoview image viewing software version 3.1.

### Biochemical analysis

To test the effect of heat on inhibition of corneal cell migration, secretomes were heated at 95 °C for 10 minutes or 65 °C for 1 hour, chilled on ice for 5 minutes and subsequently used in wound healing and cell migration assays. To determine the effect of freezing on wound healing, secretomes were independently frozen at −80 and −20 °C for one week and tested for wound inhibitory activity in cell migration assays.

To determine the approximate mass of WIF, secretomes were fractionated using molecular weight cut off (MWCO) spin columns, 3000 Da (Millipore, Amicon #UFC800324), 10,000 Da, 20,000 Da (Pierce #87751), and 30,000 Da (Amicon #UFC803008), following the manufacturers specifications. The flow through and retentate fractions were combined with LB to their original volume and tested in cell migration assays.

Chloroform extraction of secretomes was performed by placing them in an equal volume of chloroform, incubating the tubes on ice for 5 minutes, and centrifuging at 16,000 × g for 5 minutes. The aqueous and chloroform phases were harvested and the chloroform phase was air-dried using a min-vap air evaporator (Sigma Supelco #22971). Aqueous and chloroform dried samples were resuspended in LB at the original secretome volume and tested in cell migration assays as described above.

HP-20 and hydroxylapatite chromatography were performed to investigate the nature of the inhibitory factor. Five ml LB and secretomes from *S. marcescens* PIC3611 were incubated with one gram of HP 20 diaion resin (Supelco #45805) for 2 hours at room temperature with rotation. Samples were then transferred to a glass column (Biorad #737–4151) and allowed to settle for 5 minutes. LB medium (5 ml) was added to each column, allowed to settle, and then collected as the “flow through” fraction. Five ml of 100% methanol was added to each column and allowed to settle for 5 minutes, then collected and referred to as the “HP 20 elution”. Eluted samples were air-dried overnight using a min-vap air evaporator, resuspended in 5 ml LB and then tested in HCLE migration assays. Secretomes were also incubated with hydroxylapatite and prepared in the same manner as the HP-20 purifications.

### Chemical and enzymatic treatment of bacterial secretomes

LB (mock) and secretomes were exposed to 0.03 mg/ml DNase (Sigma #DN25)), 0.03 mg/ml RNase (Sigma #R6148), 0.03 mg/ml lipase (Sigma #L3126), and 0.02 mg/ml hyaluronidase (Sigma #H3506) as previously described^22^,^23^, incubated at room temperature for 1 hour, heat-treated at 90 °C for 10 minutes to inactivate the enzymes, and added to HCLEs at the same dosing ratio as with secretomes only. Secretomes were also treated with AprI (1.4 μM), a metalloprotease protease inhibitor from *Pseudomonas aerguinosa* that effectively prevents *S. marcescens* metalloprotease activity^24^ and tested for a loss of WIF in cell migration assays. All enzymes were verified as functional in control assays.

### Genetic screen for mutants unable to inhibit wound healing

The genome of *S. marcescens* PIC3611 was mutagenized using mariner transposon delivery plasmids pSC189^56^ and pBT20^57^ as previously described^58^. Colonies on LB agar were inoculated into LB in 96 well plates and grown at 30 °C overnight and frozen at −80 °C in LB containing 15% glycerol. To identify mutants unable to secrete/generate WIF, transposon mutants in 96 well plates were grown in LB at 30 °C overnight and allowed to settle for 1 day at 4 °C. Ten-microliters of transposon mutant supernatant were tested in cell migration assays supplemented with 20 μg/ml amikacin to prevent growth of transferred bacteria. Transposon insertion sites of WIF-negative candidates that were reproducibly negative for inhibiting corneal cell migration were identified using marker rescue for pSC189^56^ or arbitrary PCR^59^ for pBT20 and sequenced using primer #1880 (for pSC189 (CCTTCTTGACGAGTTCTTCTGAGC) or p241 for pBT20 (GGTTTTCTGGAAGGCGAGCATCG).

### LPS depletion experiments

To remove LPS from secretomes, polymyxin B-agarose (Sigma #P1411) endotoxin removal methods were used^46^. Five-hundred microliters of polymyxin B-agarose was washed three times in an equivalent volume of endotoxin free 0.1 M ammonium bicarbonate buffer pH 8.0. Secretomes or LB (negative control) were incubated with washed polymyxin B agarose at 4 °C with rotation for 1 hour. Samples were centrifuged at 326 × g for 2 minutes, and the supernatant was used for cell migration assays. As a negative control, sepharose beads that did not contain polymyxin B (Pharmacia Fine Chemicals #LK 00950) were used similarly to polymyxin B agarose. LPS levels in *S. marcescens* PIC3611 and polymyxin B-treated secretomes were quantified using an LAL chromogenic endotoxin quantification kit (Pierce #88282) according to manufacturer’s protocols. LB (background) was subtracted from secretome readings and LPS concentrations were graphed as EU/ml. As a validation control for the LAL assay, *E. coli* and *S. marcescens* LPS purchased from Sigma Aldrich were tested in LAL assays, and resulted in expected calculated levels of LPS.

### Effect of killed bacteria on cell migration

Overnight cultures of *S. marcescens* were normalized to OD_600_ = 2. One ml of normalized culture was treated with 50 μg/ml moxifloxacin (LKT laboratories #M5794) and 20 μg/ml amikacin for 1 hour at room temperature, incubated at 65 °C for 1 hour, and chilled on ice. Untreated and antibiotic/heat-treated bacteria were serially diluted and plated on to LB to determine colony-forming units (CFU). This treatment regimen resulted in no detectable CFU (limit of detection was 100 CFU/ml).

### LPS purification

LPS was purified by the hot phenol method according to Westphal and Jann^30^. The LPS was further purified according to Tirsoaga *et al.*  31 and quantified by LAL chromogenic endotoxin quantification assay.

### Complementation of *waaG*

The *waaG* gene (SMA4058) was amplified from *S. marcescens* K904 using primers 3507 (gaattgtgagcggataacaatttcacacaggaaacagctGCATGAAAGCATTTCGTTTGG) and 3508 (gcaaattctgttttatcagaccgcttctgcgttctgatGCAGGCCATCGATGATCATCAG) using Phusion high-fidelity polymerase (New England Biolabs). The PCR product was cloned using yeast homologous recombination^60^ under control of the *Escherichia coli P*_*lac*_ promoter shuttle vector pMQ131 (pBBR1 replicon)^60^,^61^, yielding pMQ491. The plasmid with the cloned *waaG* gene was sequenced using primer 823 (gcttccggctcgtatgttgtgtgg) to verify cloning of *waaG*. The pMQ491 plasmid was moved into the 5G1 strain that has a *waaG* mutation using conjugation for complementation analysis.

In order to clone *waaG* and the adjacent gene (*orf10*)^27^, the *waaG* gene and *orf10* was amplified from *S. marcescens* K904 using primers 3507 and 3509 (gttttatcagaccgcttctgcgttctgatTTATTGTTCTTCTTTCAGCTCAATATATTGA) amplified by PCR and cloned using yeast homologous recombination as described above yielding pMQ505. The plasmid with cloned *waaG* and *orf10* genes was sequenced with primer 823 as described above. The pMQ505 plasmid was moved into the 5G1 strain using conjugation for complementation analysis.

### Statistical Analysis

Student’s T-test and one-way ANOVA with Tukey’s post hoc statistical tests were performed using GraphPad Prism statistical software version 6.0.

## Additional Information

**How to cite this article**: Brothers, K. M. *et al.* Putting on the brakes: Bacterial impediment of wound healing. *Sci. Rep.* **5**, 14003; doi: 10.1038/srep14003 (2015).

## Supplementary Material

Supplementary Information
