# Supplementary material for: Putting on the brakes: Bacterial impediment of wound healing
Source: Sci Rep. 2015 Sep 14;5:14003. doi: 10.1038/srep14003 (PMC4650533; doi:10.1038/srep14003)
Supplement: Supplementary Information [file srep14003-s1.pdf]

Putting on the brakes: Bacterial impediment of wound healing

Authors: Kimberly M. Brothers[1], Nicholas A. Stella[1], Kristin M. Hunt[1,2], Eric G. Romanowski[1], Xinyu Liu[3], Jes K. Klarlund[1], Robert M. Q. Shanks[1,*]

[1]The Charles T. Campbell Ophthalmic Microbiology Laboratory, UPMC Eye Center, Ophthalmology and Visual Sciences Research Center, Department of Ophthalmology (OVSRC), University of Pittsburgh School of Medicine, Pittsburgh, PA.

[2]Current Address: Boston University School of Public Health, Boston MA

[3]Department of Chemistry, University of Pittsburgh, Pittsburgh, PA.

***Corresponding Author:** Robert M. Q. Shanks, EEI 1020, 203 Lothrop Street, Pittsburgh, Pennsylvania, USA 15213, Telephone: +1-412-647-3537 email: shanksrm@upmc.edu

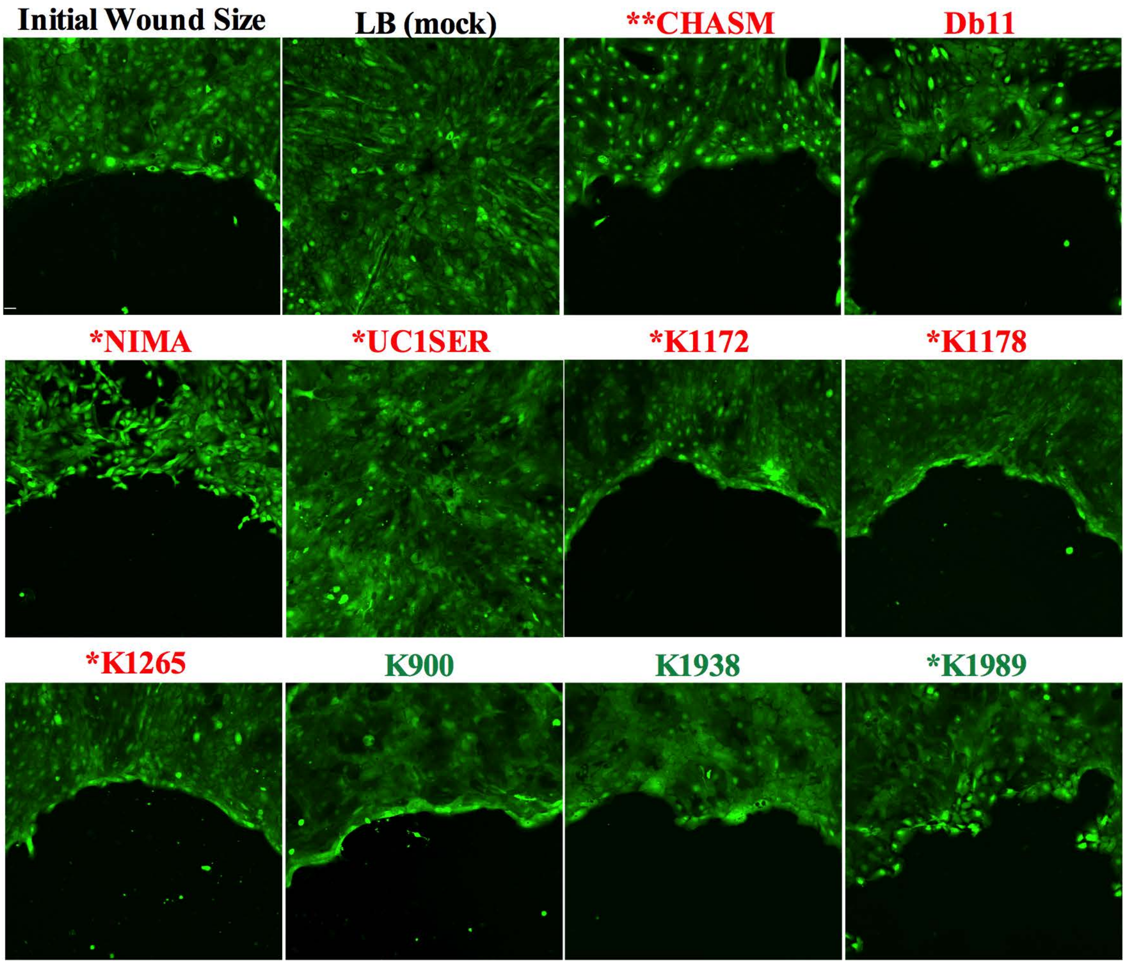

Figure S1. Secretomes from *S. marcescens* (**CHASM, Db11, NIMA, UC1SER, K1172, K1178, K1265**) and *P. aeruginosa* (**K900, K1938, K1989**) ocular clinical isolates inhibit corneal cell migration in vitro. Secretomes were prepared by diluting an overnight culture to OD_{600} = 2.0, *1.0, **0.25. LB (mock) and secretomes were added to HCLEs and incubated overnight.

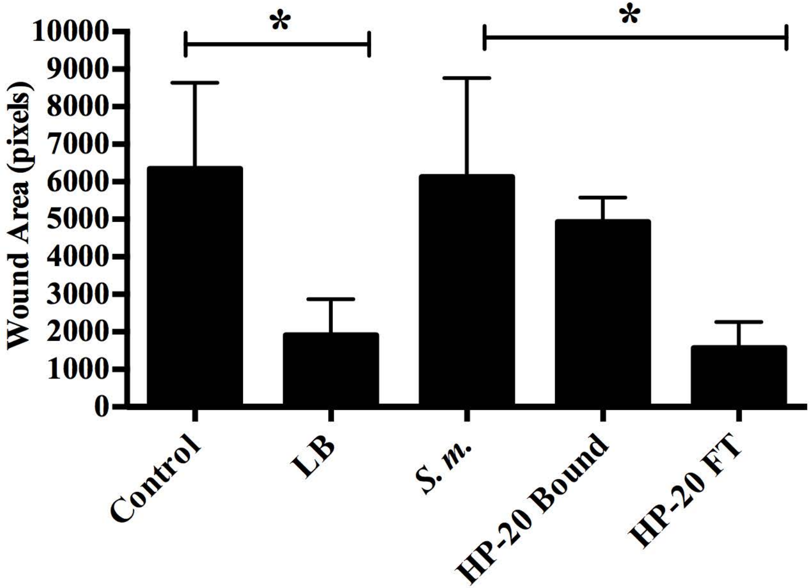

Figure S2. *S. marcescens* secretomes inhibit amoils wounded HCLE cells. Quantifcation of wound size of HCLEs treated with LB (mock) (n=5) and *S.m.* secretomes (n=5) in vitro after 48 hour incubation. Control = stratification medium alone (n=4), HP-20 bound = secretomes bound to HP-20 diaion (n=5), HP-20 FT = HP-20 diaion column flow through (n=3). Error bars = standard deviation. Error bars represent standard deviation. *p<0.01 by Tukey's post hoc analysis.

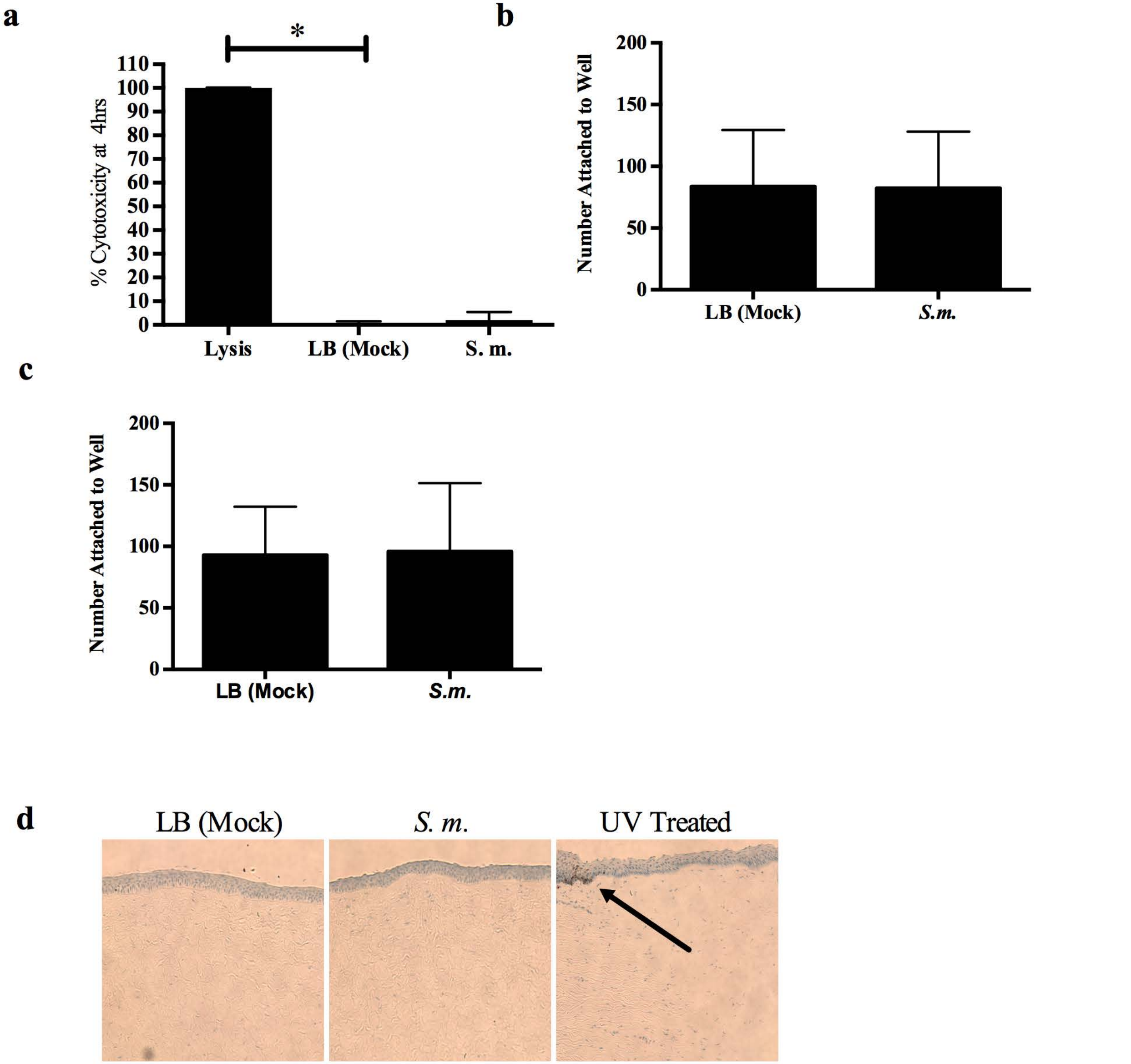

Figure S3. *S. marcescens* secretomes do not cause cytotoxicity, affect HCLE attachment, or cause apoptosis or other forms of cell death. Error bars represent standard deviation. (a) Alamar blue cytotoxicity assay of HCLEs treated with LB (mock) and *S.m.* secretomes for 4 hours. *$p<0.05$ by Tukey's post hoc analysis. (b) HCLE attachment assay to plastic tissue culture plates. LB (mock) and *S.m.* secretomes were dried on to the bottom of 12-well tissue culture dishes, or added directly to KSFM medium. HCLEs were seeded into each well and incubated for 4 hours. (d) TUNEL staining from wounded porcine corneas treated with *S. marcescens* secretomes indicating no toxicity to the tissue unless treated with UV light (Black arrow). Images are of unwounded regions of corneas.

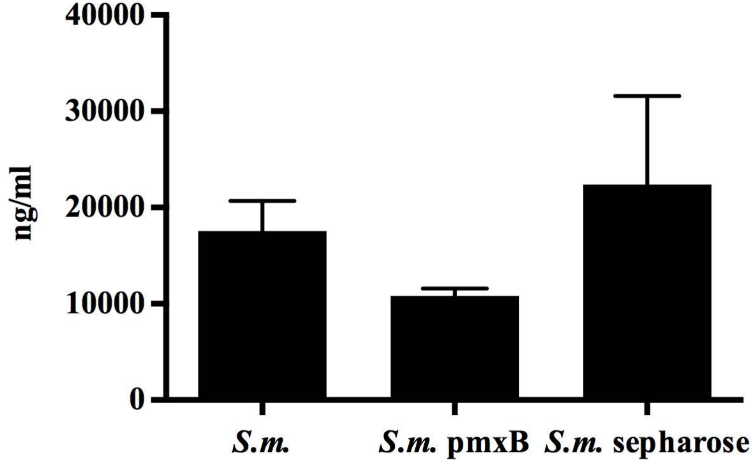

Figure S4. Quantification of LPS levels in *S. marcescens* secretomes, LPS depleted (pmxB), and and agarose bead control (sepharose) treated secretomes using LAL. Error bars represent standard deviation.

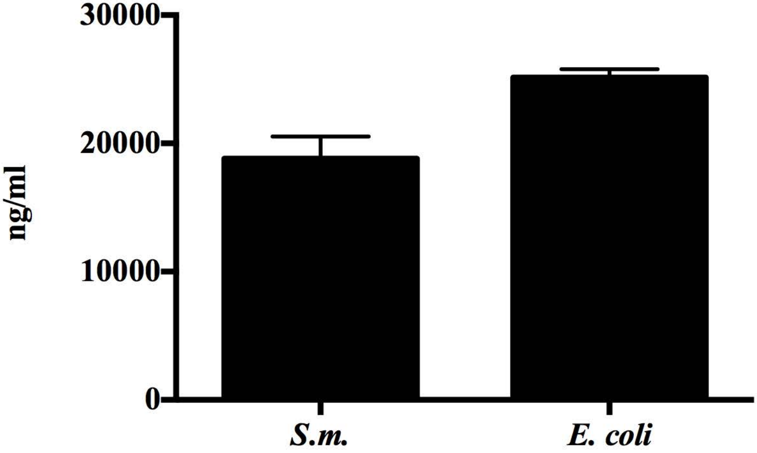

Figure S5. Quantification of LPS levels in *S. marcescens* (*S.m.*) (n =7) and *E. coli* K746 (n = 3) secretomes. *S.m.* PIC3611 secretomes had a mean of 18,814 ng/ml and *E. coli* K746 secretomes had a mean of 25,140 ng/ml as measured by LAL assay. Error bars represent standard deviation.

Supplemental Tables

Table S1. Strains and plasmids

Strain or plasmid	Description	Reference or source
S. marcescens		
CMS376	PIC3611	Presque Isle Cultures
CMS386	5G1 *waaG* transposon mutant	This study
CMS1312	*hfq* mutant	This study
CMS3842	*eepS* transposon mutant	[1]
CMS3843	*degS* transposon mutant WIF negative mutant	This study
CMS3986	*waaC* transposon mutant	This study
CMS4079	e18H12 *waaG* transposon mutant	This study
CMS4191	GNTR family transcription factor WIF negative mutant	This study
CMS4192	*gidA* pSC189 transposon mutant WIF negative mutant	This study

Table S2. Analysis of *S. marcescens* PIC3611 bacteria and secretomes

**Strain**	**Cell migration result (+ inhibited, - not inhibited)**
Amikacin killed bacteria	+
Moxifloxacin killed bacteria	+
Amikacin and moxifloxacin killed bacteria	+
Live bacteria 1.7 x 10^5 CFU	+
65°C Heat treatment 1 hour	+
95°C Heat treatment 10 minutes	-
-20°C Freezing	-
-80°C Freezing	-
Chloroform extraction (aqueous phase)	+
Chloroform extraction (Chloroform phase)	-
Ion exchange chromatography hydroxylapatite (HA)	-
Ion exchange chromatography HP-20 diaion	+

Dnase 0.03 mg/ml	+
Rnase 0.03 mg/ml	+
Lipase 0.03 mg/ml	+
Hyaluronidase 0.02 mg/ml	+
Pseudomonas aeruginosa protease inhibitor AprI 1.4 μM	+
3000 MWCO (retentate / inner chamber)	+
10,000 MWCO (retentate / inner chamber)	+
20,000 MWCO (retentate / inner chamber)	+
30,000 MWCO (retentate / inner chamber)	-

Supplemental References

1 Stella, N. A. *et al.* Serratia marcescens cyclic AMP-receptor protein controls transcription of EepR, a novel regulator of antimicrobial secondary metabolites. *Journal of Bacteriology*, **197**, 2468-2478 doi:10.1128/JB.00136-15 (2015).
